# Accuracy in Trace Analysis

**DOI:** 10.6028/jres.093.021

**Published:** 1988-06-01

**Authors:** G. Kateman

**Affiliations:** Laboratory for Analytical Chemistry, Faculty of Sciences, Catholic University of Nijmegen, Toernooiveld, 6525 ED Nijmegen, The Netherlands

Most analytical measurements are not absolute but depend on the correlation between physical phenomena and some intrinsic property, e.g., concentration. Therefore, calibration is an indispensable part of analytical chemistry. Unfortunately, calibrations are not free from interference by the environment. This disturbing environment can be the micro-environment, components in the sample that influence the calibration line. As a rule this interference is usually constant, though not always (e.g., separation processes).

The macro-environment, however, changes continuously. Temperature, pressure, chemicals, and man are stationary only during a short time. These influences will be seen as random fluctuations or, when autocorrelated, as drift. One approach is to monitor the properties of the calibration system internally by incorporating a calibration system and a measuring system. By monitoring the calibration system, the results of the unknown can be corrected. Kalivas and Kowalski [1] described the solution for the multicomponent situation, using the generalized standard addition method (GSAM). By treating drift as a time dependent component they obtain the equation

$$\Delta q_{m,l}=\sum_{s=t}^{r}\Delta n_{m,w}K_{s,l}+(V_{m}t_{m}-V_{0}t_{0})K_{t,l}$$

where

*q_m,l_*: =the volume corrected response
*n_m,s_*: = the total number of moles of component *s*
*K_s,l_*: =linear response constant for the *l*-th sensor to the *s*-th component
*m*: =number of additions of analyte
*t_m_*: =time elapsed from initial measurement up to the *m*-th addition
*V_m_*: =volume after *m*-th addition

Smit, Mars, and Kraak [2] developed a method to calibrate during a chromatographic run by applying correlation chromatography. In this technique a sample is introduced into a chromatographic column not as a single pulse, but according to a “Pseudo Random Binary Sequence” (PRBS). Smit et al. proved that it is possible to introduce more samples simultaneously, though at the expense of lengthening the PRBS sequence. Any disturbance of the system during the determination acts immediately on the calibration sample, giving a calibration factor that is obtained under ideal circumstances. Other techniques depend on prediction of the behavior of the calibration system. In this case a model is required. Assuming that drift in a calibration can be modelled as an autocorrelated random variation with known correlation constant, *Tx*, the future values can be predicted according to

$$\hat{x}(K+\tau )=\alpha^{\tau }\cdot x(K)$$

where *x*(*K*) is the normalized value of the calibration factor.

Predicting $\hat{x}(K+\tau )$ over a time span *τ* is feasible only when the predicted values do not deviate more than a preset value, *T_r_*. Müskens [3] derived that

$$\begin{array}{l} \tau =-Tx\;\ln\{Tr\;x(K)+N[x{\left(K\right)}^{2}\\ \;{-q\left(Tr^{2}-N^{2}\right)]}^{\frac{1}{2}}{\left[x{\left(K\right)}^{2}+qN^{2}\right]}^{-1}\} \end{array}$$

where $q=\left(\sigma_{x}^{2}+\sigma_{a}^{2}\right)\sigma_{x}^{-2}$, and *σ_a_*=the standard deviation of a measurement.

A powerful method which uses all available information is “state estimation” or Kalman filtering. Information from the past, or when starting the measurement from an educated guess, is used to predict the situation in the present.

$$\hat{x}(K)=F\bar{x}(K-1)-w(K-1),$$

where $\hat{x}(K)=\text{predicted state}\;\left(e.g.,\;\text{concentration}\right)\;\text{at time}\;K$, $\bar{x}(K-1)=\text{the estimation of}\;x\;\text{at}\;K-1$, *F*=the transition function, and *w*(*K*)=the variance of noise.

The next step is to do a new measurement *z*(*K*).

$$\bar{x}\left(K\right)=\hat{x}\left(K\right)+G\left(K\right)\left[\hat{x}\left(K\right)-H\left(K\right)z\left(K\right)\right],$$

where *H*(*K*)=calibration factor.

The estimate *x*(*K*) is the best estimate that is possible. The weighting factor

$$G\left(K\right)=P\left(K\right){\left[P\left(K\right)+R\left(K\right)\right]}^{-1}$$

depends on the fluctuations in the system, expressed as covariance *P*(*K*) and the measuring error *R* (K).

A Kalman filter enables the on-line estimation of calibration parameters, intercept, sensitivity and drift (of both intercept and sensitivity). The filter requires a model of the system, including system noise and measurement noise. When a good model is available, the filter can predict future values or estimate best values of the changing parameters. These figures may be used to determine when a recalibration is required.

Making a number of assumptions, the usual Kalman filter algorithms can be used resulting in [4,5,6]

$$\begin{array}{l} \hat{x}\left(K\right)=F\left(K\right)\bar{x}\left(K-1\right)\\ \hat{P}\left(K\right)=F\left(K\right)\bar{P}\left(K-1\right)F^{t}\left(K\right)+Q\left(K\;-1\right)\\ \bar{x}\left(K\right)=\hat{x}\left(K\right)+G\left(K\right)\left\{z\left(K\right)-h^{t}\left(K\right)\hat{x}\left(K\right)\right\}\\ \bar{P}\left(K\right)=\hat{P}\left(K\right)-G\left(K\right)h^{t}\left(K\right)\hat{P}\left(K\right)\\ G\left(K\right)=\hat{P}\left(K\right)h\left(K\right){\left\{h^{t}\left(K\right)\hat{P}\left(K\right)h\left(K\right)+R\left(K\right)\right\}}^{-1}. \end{array}$$

The quality performance of the system is controlled on-line by comparison with a predefined criterion. The decision to re-calibrate is followed by optimization of the concentration standards available. The example given treats drift as a deterministic phenomenon. Poulisse [7,8] indicated another approach, considering drift as a stochastic phenomenon. A novel approach aiming at the elimination of the human factor in calibration is the use of expert systems. An expert system allows the objective use of a large knowledge base. This knowledge base consists of facts and rules, the results of long experience. A so-called inference machine searches for the right solution given a set of starting parameters or states the starting parameters when the goal has been stated. Since the computer will not be annoyed when repeatedly asked for the conditions for a good calibration and can deliver calibration correction factors for each situation, it can be expected that this approach will give better calibration results. However, no conclusive results have been reported so far.
